# Giant Unilamellar Vesicle Electroformation: What to Use, What to Avoid, and How to Quantify the Results

**DOI:** 10.3390/membranes11110860

**Published:** 2021-11-07

**Authors:** Zvonimir Boban, Ivan Mardešić, Witold Karol Subczynski, Marija Raguz

**Affiliations:** 1 Department of Medical Physics and Biophysics, University of Split School of Medicine, 21000 Split, Croatia; zvonimir.boban@mefst.hr (Z.B.); ivan.mardesic@mefst.hr (I.M.); 2 Doctoral Study of Biophysics, Faculty of Science, University of Split, 21000 Split, Croatia; 3 Department of Biophysics, Medical College of Wisconsin, Milwaukee, WI 53226, USA; subczyn@mcw.edu

**Keywords:** electroformation, GUVs, cholesterol, lipid composition, lipid deposition, electrical parameters, temperature, electroformation duration, internal solution, quantitative analysis

## Abstract

Since its inception more than thirty years ago, electroformation has become the most commonly used method for growing giant unilamellar vesicles (GUVs). Although the method seems quite straightforward at first, researchers must consider the interplay of a large number of parameters, different lipid compositions, and internal solutions in order to avoid artifactual results or reproducibility problems. These issues motivated us to write a short review of the most recent methodological developments and possible pitfalls. Additionally, since traditional manual analysis can lead to biased results, we have included a discussion on methods for automatic analysis of GUVs. Finally, we discuss possible improvements in the preparation of GUVs containing high cholesterol contents in order to avoid the formation of artifactual cholesterol crystals. We intend this review to be a reference for those trying to decide what parameters to use as well as an overview providing insight into problems not yet addressed or solved.

## 1. Introduction

Artificial vesicles have become an important research tool due to their similarity to biological membranes [1,2,3,4,5,6]. Being lab-created, they enable the study of membrane properties under controlled conditions. When mimicking the biological membrane, we are interested mainly in unilamellar vesicles (only one outer bilayer), but multilamellar (bilayers arranged in concentric circles) and oligolamellar vesicles (containing smaller ones inside) can also be created. Depending on their size, unilamellar vesicles are commonly divided into three groups: small (SUVs, <100 nm), large (LUVs, 100 nm–1 μm), and giant unilamellar vesicles (GUVs, >1 μm). SUVs and LUVs are more often studied in the context of drug delivery applications [7,8,9]. GUVs are more useful as artificial cell models for eukaryotic cells due to similarity in size. Additionally, their size enables observation of membrane domain structure using light microscopy.

Here, we will focus on the electroformation method in the context of GUV formation. Although other reviews have covered similar topics, they are either not up to date [10], focus on just one segment of a specific method [11,12], or cover a much broader range of topics/methods, thus not exploring any particular approach in enough detail [13,14,15,16].

Due to a large number of electroformation parameters, it is easy to overlook some of them or underestimate the importance of a seemingly trivial step in the protocol. This motivated us to collectively analyze their impact on GUV growth in order to better understand their importance and interplay. Special emphasis is placed on GUVs with cholesterol (Chol) contents exceeding their membrane saturation limits. Membranes with such high Chol contents are of special interest to researchers investigating the involvement of Chol in the development of atherosclerosis [17,18] or to those, like our group, who investigate fiber cell plasma membranes of the eye lens [19,20,21,22,23,24,25]. High Chol and formation of pure cholesterol bilayer domains (CBDs) are signs of pathology in most tissues and organs [18,26]. Eye lenses are the only system in which such high Chol concentrations and CBDs are needed to maintain fiber cell membranes, fiber cells, and whole lens homeostasis. However, because of the high Chol content, preparation of such GUVs is problematic due to Chol demixing resulting in the formation of Chol crystals [20,27,28,29]. These crystals do not participate in further membrane formation, which leads to a real membrane Chol content that is lower than the Chol mixing ratio. We have faced these problems in our work [20] and will discuss possible ways to solve them.

The review starts by defining the classic protocol steps and then goes through each of those steps, exploring developed variations and commenting on related artifacts. Additionally, methods for quantifying the size and count of obtained vesicles are discussed. The final section goes over conclusions from the review and discusses possible improvements in the preparation of GUVs with high cholesterol contents.

## 2. Classic Electroformation Protocol

One of the earliest attempts at forming GUVs was the natural swelling method introduced by Reeves and Dowben in 1969 [3]. According to this method, a lipid solution is deposited on a surface and dried to form a lipid film. The lipid film is then rehydrated and the obtained solution gently stirred to form vesicles. The vesicles are formed mainly due to the osmotic pressure driving the aqueous solution in between the stacked lipid bilayers. Exposing the hydrophobic portion of the bilayer to aqueous solutions is unfavourable and causes them to close up into vesicles. However, the proportion of GUVs that can be generated using this method is small, as most of them are either multilamellar or display other types of defects [30]. In their efforts to devise a protocol that reliably produces a high proportion of cell-sized unilamellar vesicles, Angelova and Dimitrov applied an external electric field during lipid swelling and thus invented the electroformation method [31]. Although the exact mechanism of the method is not yet completely understood, it is believed that the electric field affects lipid swelling through direct electrostatic interactions, redistribution of counterions, changes in membrane surface and line tension, and electroosmotic flow effects [32]. More detailed theoretical discussions on the electroformation mechanism can be found elsewhere [33,34,35]; here we focus mainly on exploring the optimal parameters and artifacts appearing when performing experimental work using this method.

**Figure 1:**
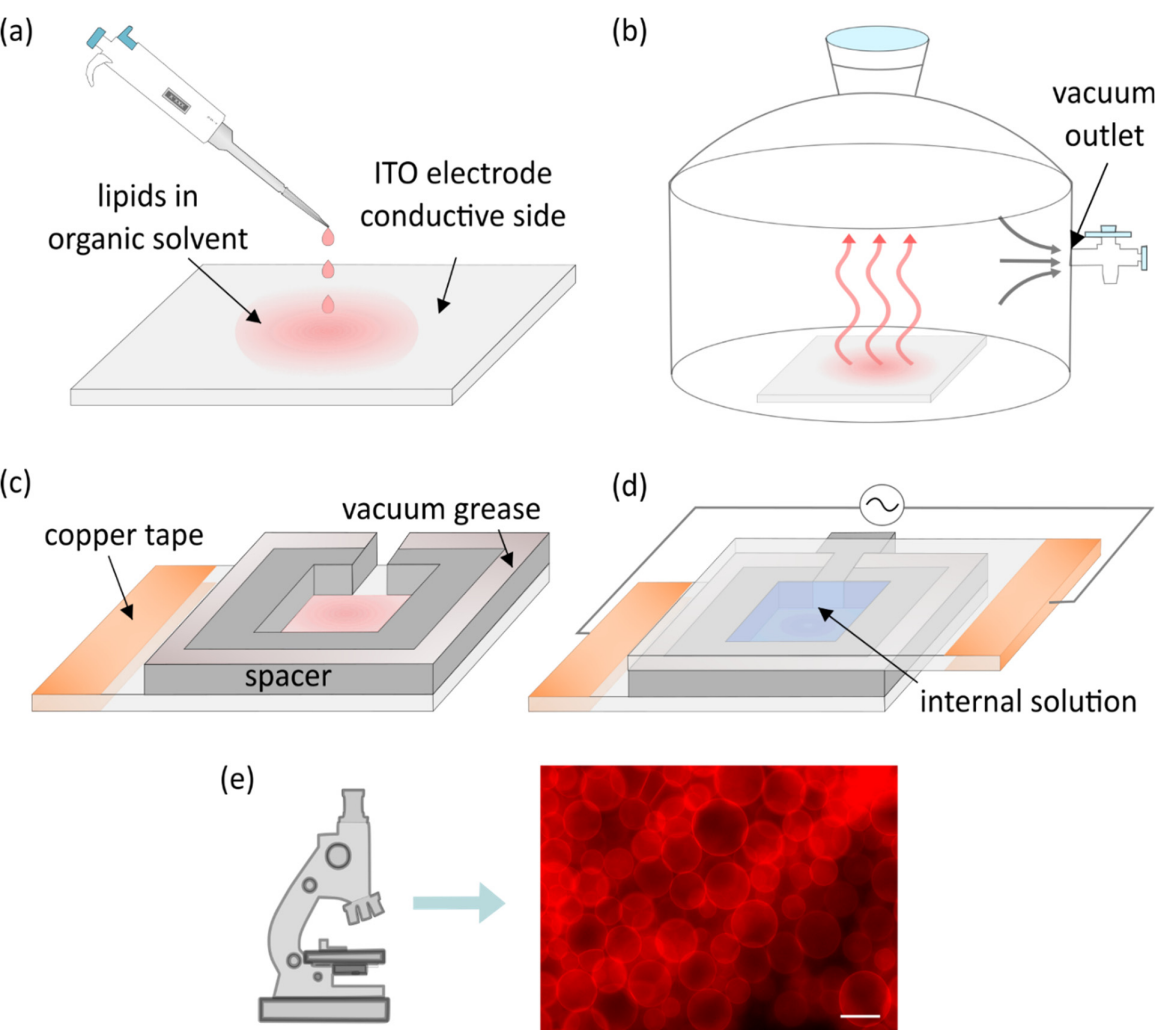
(**a**) Deposition of lipid droplets onto the electrode surface. (**b**) Evaporation of organic solvent under vacuum. (**c**) Construction of the electroformation chamber. (**d**) Electroformation chamber filled with an internal solution and connected to an alternating current function generator. (**e**) An image of fluorescently labeled giant unilamellar vesicles (GUVs) obtained using fluorescence microscopy. The scale bar denotes 50 µm.

Although Angelova and Dimitrov used platinum wires for electroformation, indium tin oxide (ITO) electrodes are most commonly used nowadays, so we utilize them in our description of the basic protocol. The only difference between these two protocols is the electroformation chamber layout. The protocol starts with droplets of lipids dissolved in an organic solvent being deposited onto the electrode (Figure 1a). In addition to lipids, fluorescent dyes are present in the mixture in small quantities to enable the usage of fluorescent microscopy later on.

The solvent is evaporated under vacuum or a stream of inert gas (Figure 1b). A spacer is attached to the electrode using vacuum grease (Figure 1c). Another electrode is then attached to the spacer with its conductive side facing inward. Following that, the chamber is filled with an internal solution of choice and connected to a voltage source while maintaining a temperature higher than the phase transition temperature of deposited lipids. Copper tape is often attached to the electrodes to provide better contact with the alternating current function generator (Figure 1d). The combination of the electric field and lipid film hydration leads to the creation of lipid vesicles which can be observed under a microscope (Figure 1e).

At first, direct current was used, but water electrolysis led to formation of bubbles [31], so a transition to alternating currents was made [31,32]. Alternating currents also introduce electroosmotic motion of the fluid, which facilitates the destabilization of the lipid film, thus promoting the formation of vesicles [32,36]. Over the years, the initial protocol has been modified in order to increase the yield, homogeneity, and compositional uniformity of the vesicles or to enable preparation of GUVs from previously incompatible lipids and buffers. In the following sections, we will systematically discuss the advancement of each segment of the electroformation method.

## 3. Electrode Materials and Cleaning

Alongside platinum electrodes, ITO-coated glass slides are most commonly used nowadays. In comparison to platinum electrodes, ITO electrodes provide a larger and flatter surface, so a higher GUV yield can be obtained. Also, owing to their transparency, microscopic techniques are easier to apply. It is recommended to periodically replace ITO electrodes since they were proven to have limited reusability. Using the same ITO glass more than three times has been shown to decrease the average GUV diameter and their quality. The effect seems to be less pronounced for zwitterionic and negatively charged lipids. Furthermore, the degradation seems to be reversible through annealing at high temperatures [37].

Compared with ITO electrodes, platinum electrode aging seems to affect the average diameter of vesicles less, but the proportion of GUVs (i.e., the proportion of defect-free unilamellar vesicles) also drops. Electrode replacement or annealing is recommended after approximately five experiments [38]. Steel syringes and copper electrodes have also been suggested as cost-friendly alternatives [39,40]. Furthermore, titanium electrodes have been advocated by some groups since their usage seems to decrease lipid peroxidation when compared to ITO electrodes [11,41,42,43].

**Figure 2:**
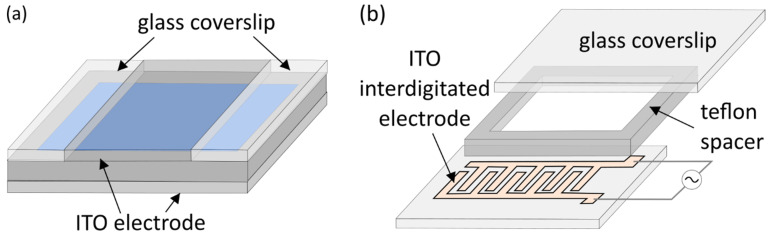
(**a**) Asymmetrical electrodes layout. The top indium tin oxide (ITO) electrode has a smaller surface than the bottom one, so it has to be surrounded by glass coverslips in order to close off the chamber. (**b**) Electroformation chamber with a coplanar interdigitated ITO electrode.

In addition to changing the electrode material completely, various modifications to the existing electrode layouts were tested. Okumura and Sugiyama confirmed that GUVs can form on non-electroconductive materials, such as polymer meshes placed onto ITO electrodes and filled with lipid solutions [44,45]. Lefrançois et al. experimented with an asymmetrical ITO electrode layout (top electrode with a smaller surface area than the bottom one) [46] (Figure 2a). This layout proved to be more efficient when physiological salt concentrations were combined with interelectrode separations smaller than 2 mm. Small interelectrode separation did not cause problems when the same parameters were set, but deionized water was used as an internal solution. The effect appears because alternating-current electroosmosis assisted flow does not occur in the symmetrical configuration. However, since both symmetrical and asymmetrical layouts were tested under the same electrical parameters, it is not clear whether GUVs could be grown under physiological conditions for smaller electrode separations just by altering the electrical parameters and retaining the symmetrical ITO chamber layout. Bi et al. abandoned the principle of parallel opposing electrodes altogether and have shown that GUVs can be grown using coplanar interdigitated ITO electrodes [47] (Figure 2b).

In order to remove contaminants from the electrode surface, researchers use a variety of cleaning protocols. In general, these consist of cleaning the electrodes using organic solvents and then drying them. Of course, there are many variations of these general steps, and some articles do not even include the cleaning protocol [29,46,48]. Variations include sonication in conjunction with organic solvents [35,47,49,50], repeated rinsing with organic solvents [51], and swabbing electrodes using lint-free wipes [10,12,28,37]. Electrodes are either dried under a stream of inert gas [35,37,47,50] or just wiped and air dried [28]. Plasma cleaning has also been tested on ITO glass [35,47] and has proved to be very effective, since it both cleans the surface and makes it more hydrophilic. Moreover, plasma cleaning could aid hydration of the solid lipid film and the formation of lipid bilayers [35].

## 4. Deposition of the Lipid Solution and Removal of the Solvent

**Figure 3:**
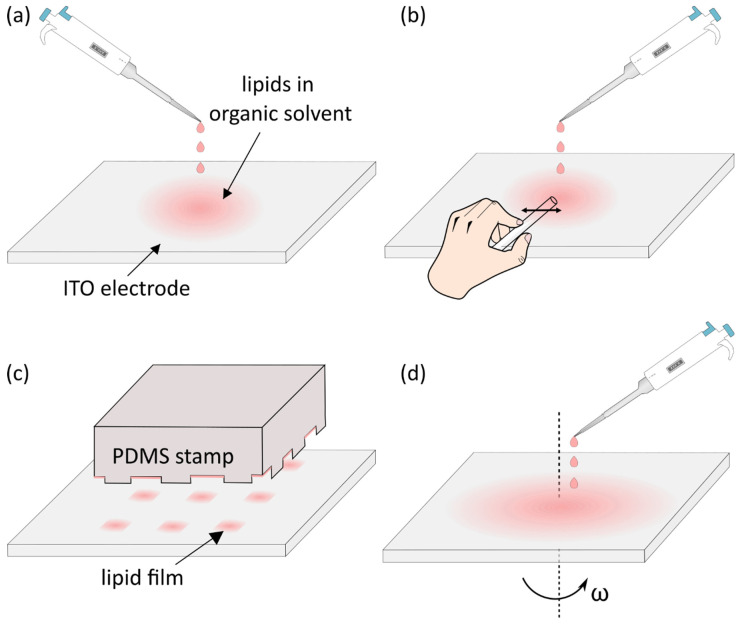
(**a**) Droplet deposition. (**b**) Droplet deposition with smearing afterward to better spread the lipid film. (**c**) Deposition of lipids by pressing a patterned silicon stamp on the electrode surface. (**d**) Spin-coating of lipid solution by fast rotation of the electrode immediately after the deposition.

In the early days of electroformation, the lipids dissolved in chloroform or other organic solvents were deposited onto the electrodes simply by dropping the solution and evaporating the solvent later [31,52] (Figure 3a). In order to efficiently produce GUVs, the lipid film is suggested to be around 5–10 bilayers thick (around 30–60 nm) [31,50]. When using drop deposition, regions of different thicknesses will be formed. Although this means that at least some portion of the deposited film will be suitable for electroformation at given parameters, other regions will inevitably be too thin or too thick. These nonuniformities lead to lack of reproducibility.

In order to make the deposition process more efficient and reproducible, different approaches were investigated as well. Some groups used needles or thin rods to smear the solution after dropping it in order to increase the homogeneity of the film [10,35,53,54] (Figure 3b). Others used a Hamilton syringe to deposit non-overlapping snakelike patterns of lipids [37]. A more systematic approach was tested by stamping the lipid solution onto the ITO electrode using a customized polydimethylsiloxane (PDMS) stamp [55] (Figure 3c). This approach resulted in GUVs of a size similar to the width of the gaps in the PDMS stamp. However, the authors reported that the thickness of the lipid film was not uniform over the lipid patches in the gaps. Dip coating (immersing the ITO electrode in the lipid solution and holding it vertically to dry) was explored as well, but due to different rates of solvent evaporation across the glass, the film proved to be inhomogeneous [50].

Reproducibly controllable lipid film thickness was achieved by Estes and Mayer using the spin-coating method. The lipid solution is dropped onto the flat electrode surface, which is subsequently rotated very fast (ω ~ 600 rpm) in order to achieve a homogenous film [50] (Figure 3d). The uniformity of films and method reproducibility were confirmed using ellipsometry and atomic force microscopy techniques. The method was proven to be effective by several groups using a wide range of lipid compositions [28,50,51]. There are, however, disadvantages as well. First, it requires a much larger amount of lipids compared with traditional methods, since much of the solution is washed away during electrode rotation [50]. Second, it involves a spin-coater, which is not a standard device in labs investigating biological membranes.

Another attempt at achieving uniform lipid films was made by Le Berre et al. [56]. They drag-dropped an organic solution of lipids on a solid substrate. Constant substrate velocity and temperature were maintained while simultaneously controlling the vapor aspiration. Reproducible results with variations of ±5 nm were obtained, but the method has not been widely adopted, probably due to the device used being relatively complicated.

Before chamber construction, the organic solvent needs to be removed from the deposited lipid solution. This is most commonly achieved by placing the electrodes under a vacuum [4,10,28,35,37,50,53]. Depending on the research group, the vacuum duration can range from just 5 min [53] to 2 h [35]. Alternatively, drying can be performed by placing the electrode with the lipid solution under a stream of an inert gas, such as nitrogen [31,51,52] or argon [41]. More rarely encountered, lyophilization can also be used to eliminate traces of the organic solvent [27,57].

Some researchers went in another direction and instead of trying to improve the existing approach, replaced the organic solution of lipids with aqueous liposome suspensions (SUVs, LUVs, or multilamellar vesicles) [4,29,49,58,59,60,61]. Using this approach, Pott et al. concluded that GUV formation was better when using aqueous liposome solutions than when using organic lipid solutions [4]. They attribute this to the ability of such dispersions to produce well-oriented membrane stacks immediately after the evaporation of water. Although any excess water was completely removed from the deposits, an alternative approach was suggested in which the deposits would be only partially dehydrated prior to internal solution addition. The effect of using damp lipid films on GUV properties was explored in more detail by Baykal-Caglar et al. [29]. By measuring their miscibility transition temperature, they have shown that such an approach produces more compositionally uniform populations of GUVs. This result is explained by a reduction in lipid demixing—a common artifact appearing when drying the lipid solution completely. The artifact is especially pronounced when using mixtures with a cholesterol content near to or above the maximum solubility threshold for that mixture [27].

Furthermore, avoiding the dry phase and use of organic solvents benefits protocols aimed at protein incorporation into GUVs, since these steps damage the protein structure. The first proof of successful protein reconstitution using this approach came from Girard et al. [61] and the most recent protocol is described by Witkowska et al. [49].

**Figure 4:**
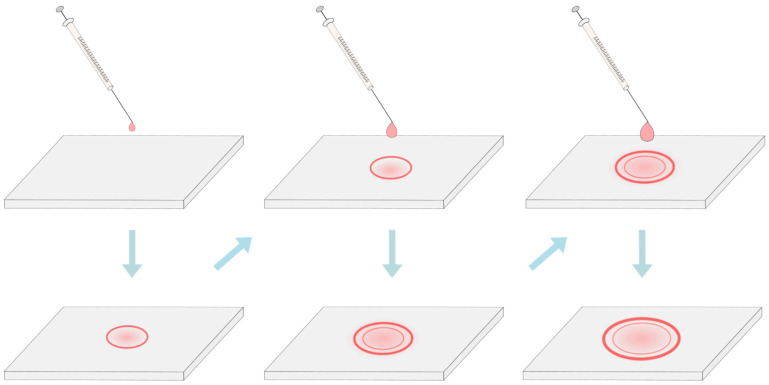
Deposition of lipids utilizing the coffee ring effect. After drying, most of the material is carried away toward the periphery and a ring-like stain is formed. This is known as the coffee-ring effect. By depositing progressively larger droplets, the ring from the previous droplets gets smeared and flattened, thus leaving behind an area of uniform lipid film thickness.

Since the deposition of aqueous liposome suspensions was a significant deviation from the classic protocol by itself, until recently, no one addressed the still existing problem of nonuniform film thickness. In 2019, Oropeza-Guzman et al. suggested using the coffee ring effect to solve this issue [59]. The effect describes a phenomenon in which a drying droplet deposits most of its material on the periphery, forming a ring-like stain. The group used the effect to their advantage by consecutively depositing progressively larger droplets on top of one another. Since a larger droplet has more material, it will create a larger diameter ring and, in the process, will smear and flatten the ring from the previous droplet, thus leaving an area of uniform lipid thickness inside (Figure 4). Although the mean diameter of vesicle populations hasn’t significantly changed when compared to the single droplet deposition, the multi-droplet preparations displayed a much lower percentage of nonunilamellar vesicles. These results combined with the low lipid mass used and a relatively simple experimental setup make the method a promising option for reproducible and uniform lipid deposition.

## 5. Lipid Compositions and Internal Solutions

Artificial vesicles are often grown from single phospholipid species. Vesicles made of different phospholipids [15,50] and hybrid phospholipid/polymer vesicles have been produced through electroformation [62,63,64]. Details about lipid/polymer vesicle electroformation can be found elsewhere [65]; here we will mainly focus on lipid composition. Among lipid mixtures, Chol and/or sphingomyelin are the most frequently employed since they are the other two most abundant eukaryotic plasma membrane components [66]. Chol concentrations of up to ~50 mol% are most commonly experimented with, since most biological membranes do not exceed this level [26,66], and the maximum solubility limit of Chol in phospholipid membranes seems to be around 66 mol% [27,67]. Nevertheless, biological systems, such as the fiber cells of eye lenses, exhibit Chol:phospholipid molar ratios of up to two in the lens cortex and up to four in the nucleus [68,69]. Excess Chol is incorporated into CBDs up to a solubility threshold after which cholesterol crystals are formed outside the membrane [22,70]. Modeling these systems requires GUVs of appropriate lipid composition, but they are not often used in experiments and additional obstacles, such as the previously mentioned demixing artifact, become more pronounced [20,27]. Phospholipid/sphingomyelin/Chol ternary mixtures have been more intensely researched of late, since these constituents are involved in the formation of lipid rafts [71].

For a long time, electroformation was deemed inappropriate for the growth of vesicles in a medium with physiological salt concentrations [72]. These difficulties have recently been linked to tighter lipid packing and decreased water permeability. Additionally, the electrohydrodynamic force decreases when using solutions with high ion concentrations [73,74]. Successful protocols for GUV electroformation under physiological conditions started appearing around 15 years ago [4,12,29,35,46,75,76]. Pott et al. successfully formed GUVs from a single lipid species in a 100–250 mM NaCl solution [4]. At their suggestion, using a similar protocol, Montes et al. also succeeded in forming GUVs from native membranes or organic lipid mixtures at physiological conditions (25 mM HEPES + 150 mM NaCl pH 7.2) [76]. Using plasma cleaned ITO electrodes, Li et al. extended the concentration of NaCl up to 2 M and noticed that the diameters of GUVs increased in the 0–200 mM range and then decreased for 200 mM–2 M. Furthermore, they successfully formed GUVs in a PBS (phosphate buffered saline) and PCR (polymerase chain reaction) buffer [35]. Two years later, another protocol focusing solely on GUV growth in a PBS buffer was published. The same article also includes a discussion on microinjection of material into grown GUVs and concludes that the highest success rate is obtained when their mechanical stability is increased through the addition of 20 mol% of cholesterol to the mixture [46].

In addition to high salt concentrations, nonneutral lipid charge also seems to affect GUV growth [35,37,53,77,78]. Estes and Mayer successfully formed GUVs from neutrally and negatively charged lipids. However, they noticed that using high concentrations of negatively charged lipids resulted in an approximately 30% increase in the thickness of the coated lipid layer [50]. Ghellab et al. and Li et al. experimented with both negatively and positively charged lipid mixtures and were able to grow GUVs using all of the combinations [35,53]. Nevertheless, the average diameter of GUVs containing higher concentrations of charged lipids was much smaller. As a possible explanation for this effect, they mention the formation of an electric double layer due to the high concentrations of counter ions near the electrode.

Alongside water and physiological buffer, GUVs are sometimes grown in sucrose and then resuspended in glucose or a physiological buffer, resulting in their sinking to the bottom of the chamber [10,12,38,41]. This makes microscopy easier since all GUVs are in the same plane. Using sugars, such as sucrose, also seems to promote GUV formation by enhancing the hydrodynamic force on the lipids through an increase in interfacial viscosity between the solution and the lipid membrane [79].

Internal solution pH is also important since it is strongly linked with lipid hydrolysis. A study using partially hydrogenated egg yolk phosphatidylcholine showed that the hydrolysis rate is lowest at around pH 6.5–7 and then increases in both directions as the pH changes [80]. This is fortunate since physiological conditions assume near-neutral pH, so those values are desirable for cell mimicking experiments.

The most common method for observation of GUVs and their domains is fluorescence microscopy. However, the user has to be careful since production of peroxides due to excitation of the fluorescent dyes leads to light-induced lipid oxidation and subsequently to light-induced domains [10,11,41,48,81]. Zhao et al. compared two dye concentrations and found that it took more time for the light-induced domains to appear for the smaller concentration (10–20 s after illumination with a mercury lamp) [48]. On top of that, fluorescent probes can change the phase behavior of membranes with a miscibility transition [10,82]. Taking the above two effects into account, it is always best to use the smallest possible amount of lipid probe while still obtaining a good signal for microscopy. Additionally, when multiple fluorescent probes are used simultaneously (for some probes even in amounts as small as 0.05 mol%), their preferential partitioning can change when compared with a single fluorescent probe scenario [82].

## 6. Electrical Parameters

Most studies are not concerned with electroformation parameters giving the best GUV yield but only require a certain number of stable vesicles for further research. It is no wonder then that most studies choose established voltage and frequency values from common references, such as Angelova et al. or Veatch, where peak-to-peak voltage is usually kept between 1 and 10 V and frequency around 10 Hz [10,31,36].

Of course, since the property of interest here is the electric field and not the voltage by itself, the voltage values should always be accompanied by electrode separation in order to provide some context for the field strength. Common interelectrode separations range from 0.5 to 3 mm. Most often, a separation value from this interval is used without special explanation, and the voltage is modified accordingly. In combination with ITO electrodes, the separation is achieved through the use of spacers usually made from silicon (PDMS) [2,31,34,37,50,51,52] or Teflon (polytetrafluoroethylene) [28,38,41,83]. Another alternative is specifying the electric field strength instead of the voltage from the beginning [4,28,51].

Significant deviations from conventional parameters are usually explored only in studies specifically trying to improve a certain aspect of the electroformation protocol. GUVs were obtained using egg phosphatidylcholine or DOPA (1,2-dioleoyl-*sn*-glycero-3-phosphate) using electric field values of up to 40 V/mm and frequencies up to 1 MHz [51]. Another study used a mixture of phosphatidylcholine and cholesterol and found the optimal values to be 5 V/mm and 10 kHz [34]. Considering such large differences depending on the specific lipid composition and experimental setup, it is prudent to optimize the electrical parameters every time a different lipid composition (or one not yet explored by other researchers) is used. However, using electric field strengths up to a couple V/mm and frequencies of 10–100 Hz seems to give satisfying results for many different lipid compositions (positive, negative, and zwitterionic lipids compositions were tested) when deionized water is used as an internal solution [53].

If physiological salt concentration buffers are used as the internal solution, higher frequencies tend to be needed [4,12,35,76]. Pott et al., Montes et al., and Lefrancois et al. all had success forming vesicles at physiological conditions using a frequency of 500 Hz [4,46,76]. Li et al. performed a systematic frequency-voltage sweep for three lipid compostions (two zwitterionic and one negatively charged lipid species) and found the ideal frequency to be in the range of 100–1000 Hz as well [35]. The preference for higher frequencies is attributed to disruption of the electric double layer under those conditions [12,35,51,84].

Some groups alter the voltage values during electroformation [4,38,39,46,49,54,61,76]. The reasoning behind such protocols is well described by Pott et al. [4]. First, the electric field is progressively being increased up to a maximum value at a fixed frequency in order to maintain a sphere-like shape of growing vesicles. Second, depending on the chamber solution and the duration of the first step, the electric field is kept constant in order to allow an increase in size through swelling. An additional step can be included in which the electric field remains the same as in the previous step, but the frequency is reduced in order to promote vesicle fusion and detachment from the electrodes.

Drabik et al. compared the approach of increasing the voltage by 1 V every hour from 1 to 4 V with a constant 2.5 V applied for 4 h. The vesicles obtained using the sequential voltage approach were bigger, but both the amount of lipids used for electroformation and the ratio of unilamellar to oligolamellar vesicles remained unchanged [38]. It is important to note that this research only used a zwitterionic monounsaturated phospholipid, so the results might differ when using different lipids or more complex compositions. Breton et al. utilized a protocol involving an initial stepwise increase in voltage up to a maximum value that was then maintained for a certain amount of time [85]. Using three lipid species with different levels of unsaturation, they have shown that even at relatively low electric field values (<1 V/mm), lipid oxidation occurs for moderately and highly oxidizable lipids (two double bonds or more). This is in agreement with a previous study on the oxidative effect of electric field on lipids from Zhou et al. [42]. The increase in maximum voltage did not change the rate of oxidation for monounsaturated lipid species [85]. The size of the vesicles increased up to an oxidation level of 25%, after which it started to decrease [85]. The fact that oxidation of 25% will be reached at different voltages for different lipid species again underlines the necessity of optimizing the protocol for each different lipid composition used.

## 7. Temperature and Electroformation Duration

Temperature is an important parameter in electroformation protocols since it governs the bilayer gel to liquid phase transition (melt/transition temperature). Moreover, a continuous increase in temperature (as opposed to a thresholding effect of the transition temperature) seems to be accompanied by an increase in the final diameter of produced GUVs [35,47,53]. A probable explanation is that the temperature enhances the permeability of the membrane to the solvent through an increase in membrane fluidity [86]. Although increasing the GUV diameter is a desirable property, there is a disadvantage to increasing the temperature too much since prolonged exposure to high temperatures leads to increased lipid breakdown [10].

In order for the bilayer to be in the liquid phase and adequate mixing of components to be achieved, the electroformation temperature is usually kept above the transition temperature of the lipid with the highest transition temperature in the mixture. In some cases, the miscibility transition temperature can even exceed this highest transition temperature, so an even higher value is sometimes needed [10]. However, high temperatures can be harmful in scenarios such as those involving protein reconstitution. Regarding this issue, a study using a DOPC (1,2-dioleoyl-*sn*-glycero-3-phosphocholine)/sphingomyelin/Chol (2:2:1 molar mixture) compared the properties of GUVs grown at room temperature and 65 °C [83]. The proportion of liquid ordered–liquid disordered (Lo + Ld) phase separated vesicles was higher in the higher temperature batch. Nevertheless, the physical properties of vesicles that were phase separated were similar for both temperatures.

The rate of sample cooling after electroformation plays a significant role in the phase separation of membrane domains. First, sudden changes in temperature can break some vesicles [10]. Second, if cooled too quickly from a fluid one-phase region to the gel–fluid phase, the vesicles seem to be caught in a nonequilibrium state where phase separation has not yet been achieved [87,88]. On the other hand, in situations where the Ld phase is present in small amounts, cooling too slowly can lead to an artifactual decrease in the amount of Lo + Ld phase due to the Ld phase pinching off the parent GUV [11].

The duration of electroformation is variable between research groups as well and usually ranges from 0.5 to 7 h, though it can last up to 24 h in some cases [38]. The effect of prolonged electroformation duration (4–24 h) has been tested using fluorescence microscopy, flow cytometry, spectrofluorimetry, and colorimetric analysis to measure the diameter of vesicles, the proportion of oligolamellar vesicles, and the amount of lipid molecules in the GUV suspension after electroformation [38]. Prolonged electroformation duration did not significantly impact any of these parameters. However, the study only used a monounsaturated phospholipid, which is poorly oxidizable. Another study involving three lipids with different degrees of unsaturation was also conducted [85]. Using mass spectrometry and flow cytometry, a longer electroformation duration (5 vs. 20 h) was shown to induce lipid oxidation in more oxidizable lipids (two double bonds or more).

## 8. Vesicles Population Count and Size

### 8.1. Manual Analysis

Electroformation results are usually presented through the number and size of analyzed vesicles. The most common approaches use microscopic techniques and then count the vesicles and measure the diameters or intensities manually. Of course, since the number of GUVs in a chamber can be very high, it is not feasible to analyze every vesicle, so usually, a predetermined number of GUVs is selected. Unfortunately, this process is highly subjective and can lead to problems, such as biased selection or different criteria for selection. Consequently, there has been a recent surge in the development of automatic methods for GUV analysis. Based on the operating principle, methods for automated GUV detection and analysis can be divided into three larger groups: conventional microscopy aided by automatic detection algorithms, light scattering methods, and methods based on analysis of electrical impedance.

### 8.2. Automated Microscopy Analysis

Compared with manual analysis, automatic analysis can save a lot of time and reduce the possibility of bias while tracking the vesicle features. Nevertheless, many research groups still default to manual analysis, so studies using automated approaches do not appear often and are generally published within last 10 years. Numerous different approaches were used, such as circle detection using discrete differential evolution [89], Markov random field [90], and circular Hough transform [91]. The latest additions are offered as ImageJ [92] macros using (amongst others) techniques such as polar transformations [93,94] or a series of steps sometimes utilizing existing ImageJ functionalities [95,96].

3D reconstruction algorithms based on confocal microscopy stacks are available as well [97]. Although a 3D reconstruction is visually the best approach, we can never display the whole surface at once. In order to gain a complete information, we have to use multiple viewing angles. This problem can be solved by creating 2D projections from 3D image stacks through unfolding of the 3D surface (like projecting Earth’s surface on a cartographic map) [98]. Additionally, since the 2D projection displays the whole surface at once, it is much easier to track the surface dynamics. Maybe the best approach would be to use these two methods together, since they complement each other; one gives us a familiar and visually appealing 3D representation and the other a possibility to monitor the whole surface at once.

### 8.3. Light Scattering Methods

Light scattering methods are a popular tool for determining the average diameter of particles suspended in a solution. For particles up to a diameter of around 1 μm, methods such as dynamic light scattering can be used to easily obtain the average diameter value [99]. Since GUVs fall out of this size category, the most commonly used light scattering method is flow cytometry [38,85,100,101,102,103,104]. Studies have been performed to confirm the correlation between the forward scatter (FSC) and side scatter (SSC) signal intensity and particle size. For example, the correlation for smaller particles (~1 µm) has been confirmed using dynamic light scattering measurements [99] and for larger ones (~10 µm) by observing liposomes under a microscope after fluorescence-activated cell sorting [101]. Consequently, to obtain the average liposome diameter, sample FSC and/or SSC signals are measured and compared with those of calibration beads (usually polystyrene or silica) of known size.

The problem with this approach is that the FSC and SSC signals depend on the internal structure of the particles as well as their refractive indices, giving rise to a certain amount of error in obtained diameters [99,105]. Usually, researchers are content with obtained relative sizing but comment on the possibility of error due to the refractive index mismatch. Possible solutions have been offered to account for the difference. One study first obtained the average refractive index of particles of interest and then corrected the calibration beads measurements using the Mie scattering theory [105]. Another study took a more direct approach and proposed artificial liposomes as calibrators instead of polystyrene beads [100]. An additional detail which is easily missed is that all of the articles above use FSC and/or SSC to extract the dimensions of the particles. However, when particles are approximately the same size as the height of the laser beam (as is the case for most GUVs), pulse width sizing is probably a better approach for determining the size of vesicles as it does not depend on many of the factors plaguing light scattering approaches for size measurements [106,107].

Recently, imaging flow cytometry devices started being used for GUV quantification [103]. This unites the best of both worlds by combining the rapid analysis of thousands of particles using flow cytometry with the capability of image-based approaches to identify the results.

### 8.4. Electrical Impedance-Based Analysis

Coulter counters detect particles suspended in an electrolyte based on the change of impedance due to the passing of a particle through an aperture between the two electrodes [108]. In terms of potential for analysis of GUVs, the method has an advantage of being able to detect the size of particles as well as their number.

With this possibility in mind, it is surprising that this device is not used more in GUV studies, so it is probably due to the device not being widely accessible. We were able to find only one recent GUV study using this method [109]. Even there, however, not a classic Coulter counter but a handheld version was used. Although combining the principle with a handheld device is convenient, the drawback is that it can only be used to analyze particles ranging from 6 to 36 μm in diameter. However, depending on the aperture size, a classic Coulter counter can be adjusted to analyze objects as large as 1600 μm [108].

## 9. Final Conclusions and Future Directions

This section summarizes the most important modifications of the electroformation method and discusses future improvements regarding the formation of GUVs with a high Chol content.

Concerning electrodes, ITO and platinum remain the most common choices, but the same electrodes should not be used for too long due to the negative impact of electrode aging on GUV quality [37,38]. Traditional drop-deposition methods have been superseded due to their not being able to attain reproducibly uniform lipid films. The best alternatives seem to be the spin-coating method of Estes and Mayer [50] and deposition using the coffee-ring effect [59]. Of these two, the coffee-ring method offers the additional advantage of requiring fewer lipids. Replacing the organic solution of lipids with an aqueous liposome suspension during the deposition step has also been shown to improve GUV formation [4]. Bypassing the dry phase altogether by using damp lipid films for electroformation has been tested as well and shown to produce more compositionally uniform GUV populations [29]. The effect is attributed to a reduction in the lipid demixing artifact that occurs when the lipid film is dried completely.

In order to better emulate cell conditions, solutions with physiological pH and ion concentrations have to be used instead of deionized water. Using plasma cleaned ITO electrodes and higher electroformation frequencies (~500 Hz) seems to be beneficial for GUV growth in such environments [4,35,46,76]. Depending on the degree of unsaturation of used lipids, the voltage should be kept low enough to prevent oxidation. For highly oxidizable lipids, oxidation occurs even at relatively low field values such as 1 V/mm [85]. Overly long electroformation durations should also be avoided since they too increase lipid oxidation for polyunsaturated lipids [85].

Electroformation temperature should be kept well above the transition temperature of the lipid with the highest transition temperature in the mixture. Increasing the temperature has been shown to lead to an increase in final GUV diameter [35,47,53]. Nevertheless, temperature should not be set too high since prolonged exposure to high temperatures leads to increased lipid breakdown [10]. The rate of cooling should be monitored as well. Cooling too quickly can lead to vesicle rupture [10] and vesicles being caught in a nonequilibrium state [87,88]. However, if cooling occurs too slowly in situations where Lo + Ld phases are present, the Ld phase can pinch off the parent GUV, leading to artifactual results as well.

The most common method of quantifying vesicle yield is fluorescence microscopy followed by manual vesicle analysis. However, this approach can be highly subjective, leading to biased results. Consequently, numerous automated algorithms for GUV detection have recently been developed and implemented [89,90,91,93,94,95]. Aside from traditional image-based analysis, flow cytometry and impedance-based methods have also been used to assess the GUV yield. Flow cytometry provides information on both the size and structure of the analyzed population. However, the size measurements may be incorrect due to the cytometry signal dependance on the particle structure and refractive index [99,105]. Impedance-based analysis offers much more precise size measurements but lacks the additional information flow cytometry offers. Recently, imaging flow cytometry was applied to GUV analysis, combining the rapid population analysis of flow cytometry with the ability to identify the results through their images [103].

Applying classic electroformation protocols to produce GUVs from lipid mixtures containing high Chol concentrations leads to artifacts caused by Chol demixing and formation of Chol crystals. We faced these problems using confocal microscopy to confirm that pure CBDs are formed in GUVs made of a Chol/PL mixture [20]. We were able to observe CBDs but only when the Chol/PL mixing ratio was equal or greater than 2.5 for DSPC (distearoylphosphatidylcholine) and 3 for POPC (1-palmitoyl-2-oleoyl-glycero-3-phosphocholine). We previously showed that for these phospholipids CBDs start to form at 50 mol% Chol concentration in lipid bilayer membranes of multilamellar liposomes (at a Chol/PL molar ratio of 1) [22,70]. At a membrane Chol content of 66 mol% (i.e., at a Chol/PL molar ratio of 2), Chol crystals start to form [22,70]. Thus, the real Chol content in the membranes of GUVs was significantly lower than the Chol/PL mixing ratio in the chloroform solution used for GUV preparation. The multilamellar liposomes were prepared using the rapid solvent exchange method [22,70], which protects against the demixing of Chol in the form of Chol crystals. Classic electroformation protocols contain steps of the film deposition method (see Section 2). During membrane preparation, using the film deposition method, the lipid mixture passes through the solid-state intermediate at which solid-state demixing of Chol in the form of Chol crystals take place. Chol crystals do not participate in the further membrane formation, resulting in a real membrane Chol content lower than the Chol mixing ratio [27]. This problem in the formation of GUVs could be solved by replacing the organic solutions of lipids with aqueous suspensions of compositionally uniform liposomes formed using the rapid solvent exchange method [22,110,111]. These liposome suspensions can then be used to form damp lipid films for electroformation. Baykal-Caglar et al. tested this approach, but only for liposome suspensions with lower Chol concentrations (up to ~40 mol%) [29]. We think that this approach can be extended to higher Chol contents reaching Chol saturation limits and Chol solubility thresholds.

## Data Availability

Not applicable.
